# Ibuprofen and ketoprofen potentiate UVA-induced cell death by a photosensitization process

**DOI:** 10.1038/s41598-017-09406-8

**Published:** 2017-08-21

**Authors:** Emmanuelle Bignon, Marco Marazzi, Vanessa Besancenot, Hugo Gattuso, Guillaume Drouot, Christophe Morell, Leif A. Eriksson, Stephanie Grandemange, Elise Dumont, Antonio Monari

**Affiliations:** 1 Institut des Sciences Analytiques, UMR 5280, Université de Lyon1 (UCBL) CNRS, ENS Lyon, Lyon, France; 2 Université de Lyon, ENS de Lyon, CNRS, Université Lyon 1, Laboratoire de Chimie UMR 5182, F69342 Lyon, France; 3 Theory-Modeling-Simulation, Université de Lorraine − Nancy, SRSMC, Boulevard des Aiguillettes, Vandoeuvre-lès-Nancy, Nancy, France; 4 Theory-Modeling-Simulation, CNRS, SRSMC, Boulevard des Aiguillettes, Vandoeuvre-lès-Nancy, Nancy, France; 5 CRAN, UMR 7039 Université de Lorraine-Nancy, Vandoeuvre-lès-Nancy, Nancy, France; 6 CRAN, UMR 7039 CNRS, Vandoeuvre-lès-Nancy, Nancy, France; 7 Department of Chemistry & Molecular Biology, University of Gothenburg, Medicinaregatan 9 c, 40530 Göteborg, Sweden

## Abstract

Nonsteroidal 2-arylproprionic acids are widely used, over-the-counter, anti-inflammatory drugs. Photosensitivity is a commonly overlooked adverse effect of these drugs. Based on the combined use of cell viability assays and molecular modeling, we prove and rationalize the photochemical pathways triggering photosensitization for two drugs, ibuprofen and ketoprofen. As its parent compound benzophenone, ketoprofen produces singlet oxygen, upon triplet manifold population. However, ibuprofen and ketoprofen photodissociate and hence may generate two highly reactive radicals. The formation of metastable aggregates between the two drugs and B-DNA is also directly probed by molecular dynamics. Our approach characterizes the coupled influence of the drug’s intrinsic photochemistry and the interaction pattern with DNA. The photosensitization activity of nonsteroidal 2-arylproprionic acids, being added to gels and creams for topical use, should be crucially analyzed and rationalized to enact the proper preventive measures.

## Introduction

**Figure 1:**
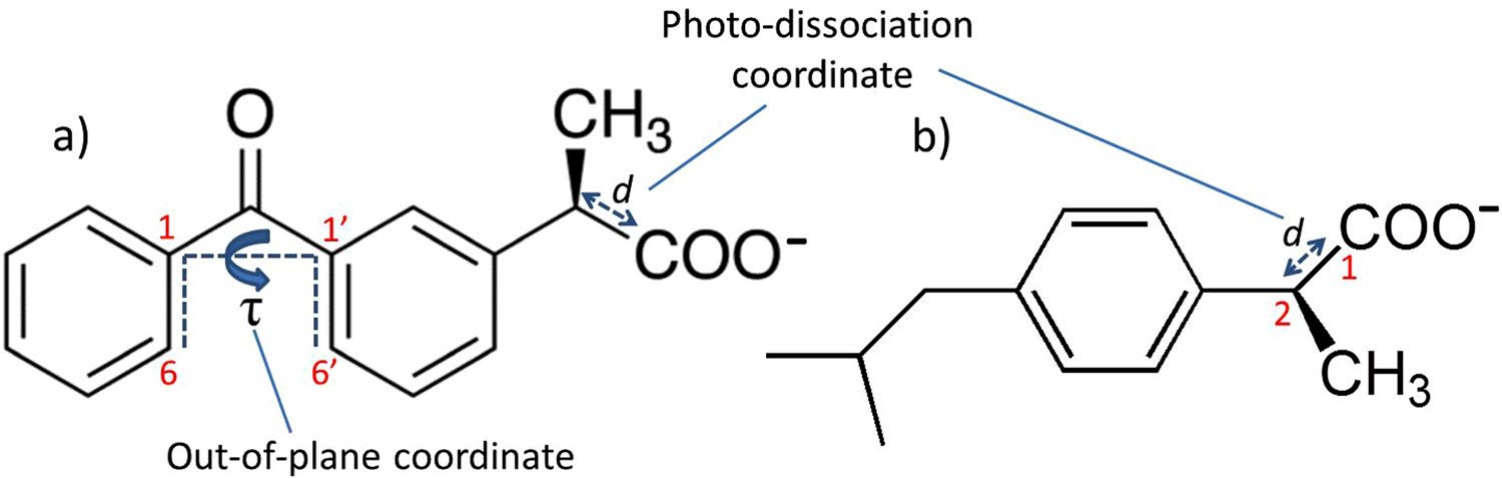
Molecular structures of ketoprofen (**a**) and ibuprofen (**b**). The two coordinates *d* and τ leading to the two different photophysical pathways are also depicted.

Ibuprofen^1–5^ and ketoprofen^6–8^ (Fig. 1) are two common nonsteroidal anti-inflammatory drugs (NSAIDs) that are used since many years due to their anti-inflammatory^9^, analgesic^10^, and antipyretic^11, 12^ properties. As many other NSAIDs^13–15^ they inhibit the cyclooxygenase enzyme^16^ and act by decreasing the production of prostaglandine inflammatory precursors. Although efficient, both drugs present known side effects in particular related to the insurgence of stomach or intestinal bleeding^17–19^ and of circulatory or cardiac deregulation^20–22^. Ibuprofen was also associated to the possible appearance of skin blistering^23, 24^ and photosensitizing activity was recognized for both NSAIDs^25–28^ also leading to photohemolysis in the case of ketoprofen^29^. Both drugs are commonly sold by prescription or as components of other formulations also available over the counter, the sales volumes being impressively high. Most notably, both ketoprofen and ibuprofen can be found as key components of anti-inflammatory topical gels or creams to be applied on the skin^30–33^, and ibuprofen is also associated with acne treatment^34^.

Ketoprofen is a 2-arylpropionic acid derivative, featuring a benzophenone core (Fig. 1). The latter compound, known for its photo-reactivity and photocatalysis efficiency, is usually regarded as a paradigmatic photosensitizer in particular acting against DNA^35^. A photosensitizer is a chromophore whose light absorption triggers photophysical or photochemical phenomena leading to the direct or indirect damage of biological macromolecules^36–38^. Photosensitization mechanisms may involve energy-^39, 40^ or electron-transfer^41–44^, or activation of molecular oxygen^45, 46^ via production of the reactive singlet oxygen (^1^O_2_) that ultimately will produce oxidative reactions. Benzophenone has been shown to act via all the different mechanisms^35, 47, 48^, and due to the relatively efficient population of its triplet manifold, triplet-triplet energy transfer or^1^O_2_ activation are preferred. However, the phototoxicity of NSAIDs is far less established and should be properly characterized. In this contribution, we rationalize the photosensitization efficiency of ketoprofen performing cell viability assays over different cell lines exposed to ketoprofen and UVA light. We also unveil the photochemical mechanisms at atomic resolution, and clarify the interactions with biological macromolecules and DNA in particular^36^.

The same protocol and procedure are applied to ibuprofen, to also trace back its reported but non-clarified photosensitizing action^25, 26, 49^. For this drug, commonly used for topical skin applications^50^, the molecular similarity with known sensitizers is much less pronounced. Hence one cannot a priori surmise the photosensitizing efficiency nor hypothesize an evident photochemical pathway. Previous computational studies have revealed complex photochemical pathways for ibuprofen^51, 52^ and ketoprofen^53^, mainly involving dissociative mechanisms. Combining cell biology techniques and state-of-the-art molecular modeling, we confirm the sensitizing activity of both drugs under UVA absorption. The unraveling, and direct comparison, of the two very different sensitization pathways provides a rationale for the differential effects on cell viability. Furthermore, protective strategies to avoid unwanted sensitization may also be envisaged based on the results of the present communication.

## Materials and Methods

### Cell culture and treatment

MCF7 and MDA-MB231 (ATCC) breast cancer cell lines were cultivated in RPMI 1640 (Gibco) supplemented with 10% of fetal calf serum (Sigma), 2 mM of L-glutamine (Sigma), and 0.1 mg/mL of gentamycin (Sigma) at 37 °C, 5% CO_2_.

Cell treatment: ibuprofen (K1751, Sigma) and ketoprofen (I4883, Sigma) were solubilized in ethanol at 200 mM and 50 mM concentrations, respectively. Cells were cultivated for 24 h and treated with 500 µM of ibuprofen or ketoprofen for 24 h, note that the chosen concentration of the drug is much lower, by some order of magnitudes, than the one present in ibuprofen topic creams. Control cells were treated for 24 h with only ethanol, after cultivation. After the treatments, cells were submitted to UV (0.5µJ/s/cm^2^) irradiation at 302 nm during 15 or 30 seconds and re-incubated for 17 h (MCF-7) or 6 h (MDA-MB231) before analyses.

### Cell viability, MTT assay

Cells were plated with 2.10^5^ cells per well in 3.5 cm plates for 24 h with 2 ml of culture medium and treated as mentioned above. Cell viability was evaluated using MTT (3-[4,5-dimethylthiazol-2-yl]-2,5- diphenyltetrazolium bromide) staining. Afterwards, the medium was replaced by a solution containing 0.5 mg/mL of MTT and incubated 2 h at 37 °C. Yellow Tetrazolium (MTT) is converted, by metabolically active cells, to an insoluble purple formazan that is then dissolved by adding 0.5 mL of SDS 25%. Absorbance measurements were performed at 570 nm (plate reader, Victor^TM^ × 3 Perkin Elmer). Results are expressed as percentage of cell viability compared to control cells (corresponding to 100%).

### Apoptosis detection, western blot

After drug treatments, detached and attached cells were collected and lyzed with lysis buffer (TrisHCl 10 mM pH 7.4, EDTA 5 mM, Triton × 100 1%, protease inhibitor cocktail (Roche)) for 20 min on ice. Protein concentration was determined by the Bradford method and 50 µg of protein were loaded on 10% SDS-PAGE gels and transferred to PVDF membrane. Membranes were blocked with 5% non-fat milk in TBST buffer (20 mM Tris-HCl, 120 mM NaCl, 0.1% Tween 20) for 1 h and then incubated with a cleaved-PARP primary antibody at 1/1000 (552596, BD Biosciences) and tubulin at 1/2500 (AB52866, Abcam) overnight at 4 °C. After washing, membranes were incubated with HRP-coupled secondary antibody for 1 h and signals were revealed with a chemiluminescence system (Biorad) and recorded with the Chemidoc Touch system (Biorad). Densitometric analysis was performed using Image J software.

### Statistical Analysis

All results are represented as mean value ± standard error of mean (SEM). Statistical analyses were performed by using 2-way Anova test with Bonferroni posthoc (GraphPad Prism) allowing a comparison between: i) UV effects, ii) ibuprofen or ketoprofen effects and iii) UV plus ibuprofen or ketoprofen effects. Statistically significant results were represented as follows: *p < 0.05, **p < 0.01, ***p < 0.001.

### Quantum chemistry

The *ab initio* multiconfigurational CASPT2//CASSCF methodology^54, 55^ and time dependent-density functional theory (TD-DFT)^56, 57^ were applied, using the Molcas 8^58^ and Gaussian 09^59^ programs, respectively. In the CASPT2//CASSCF calculations, active spaces of 10 electrons-in-9 orbitals and 12 electrons-in-11 orbitals were considered for ibuprofen and ketoprofen, respectively, coupled to the ANO-L-VDZP basis set. For both molecules, the CAM-B3LYP^60^/6-311 + G(d,p) level of theory was applied for the TD-DFT calculations, after benchmarking different functionals (see Supplementary Information). The CAM-B3LYP functional was chosen in order to assure a balanced description of local and charge-transfer excited states. In all cases, water solvent was modeled by the polarizable continuum model (IEF-PCM)^61^. TD-DFT spin-orbit couplings^62^ were calculated with the Dalton2016^63, 64^ suite of programs. At the TD-DFT level of theory, the electronic density reorganization of the excited states was analyzed in terms of natural transition orbitals (NTO)^65^ obtained with the Nancy_EX code^66^.

### Molecular Dynamics

All molecular dynamics simulations were carried out with the AMBER12 program^67^. The 10-bp poly(A-T) oligonucleotide was built using the *NAB* module. DNA force field parameters were taken from parm99^68^ with bsc1 corrections^69^. Ibuprofen and ketoprofen atomic point charges were computed following the RESP protocol, while parameters were generated using the GAFF force field^68^. The solvent was modeled by a TIP3P^70^ water box. Starting structures of the DNA/NSAID complexes were taken from a former study of benzophenone interactions with DNA^71^. Simulations were performed for several initial orientations of the drug interacting with DNA (8 for ketoprofen and 4 for ibuprofen) exhibiting either insertion or minor groove binding. After equilibration, trajectories of up to 300 ns were obtained at a temperature of 300 K (see Supplementary Information for more details).

## Results

To determine the biological effect of UV exposure (that is by itself an important death inducer), on ibuprofen and ketoprofen pretreated cells, their viability was evaluated by measuring metabolically active cells that are supposed to reflect their intrinsic viability.

**Figure 2:**
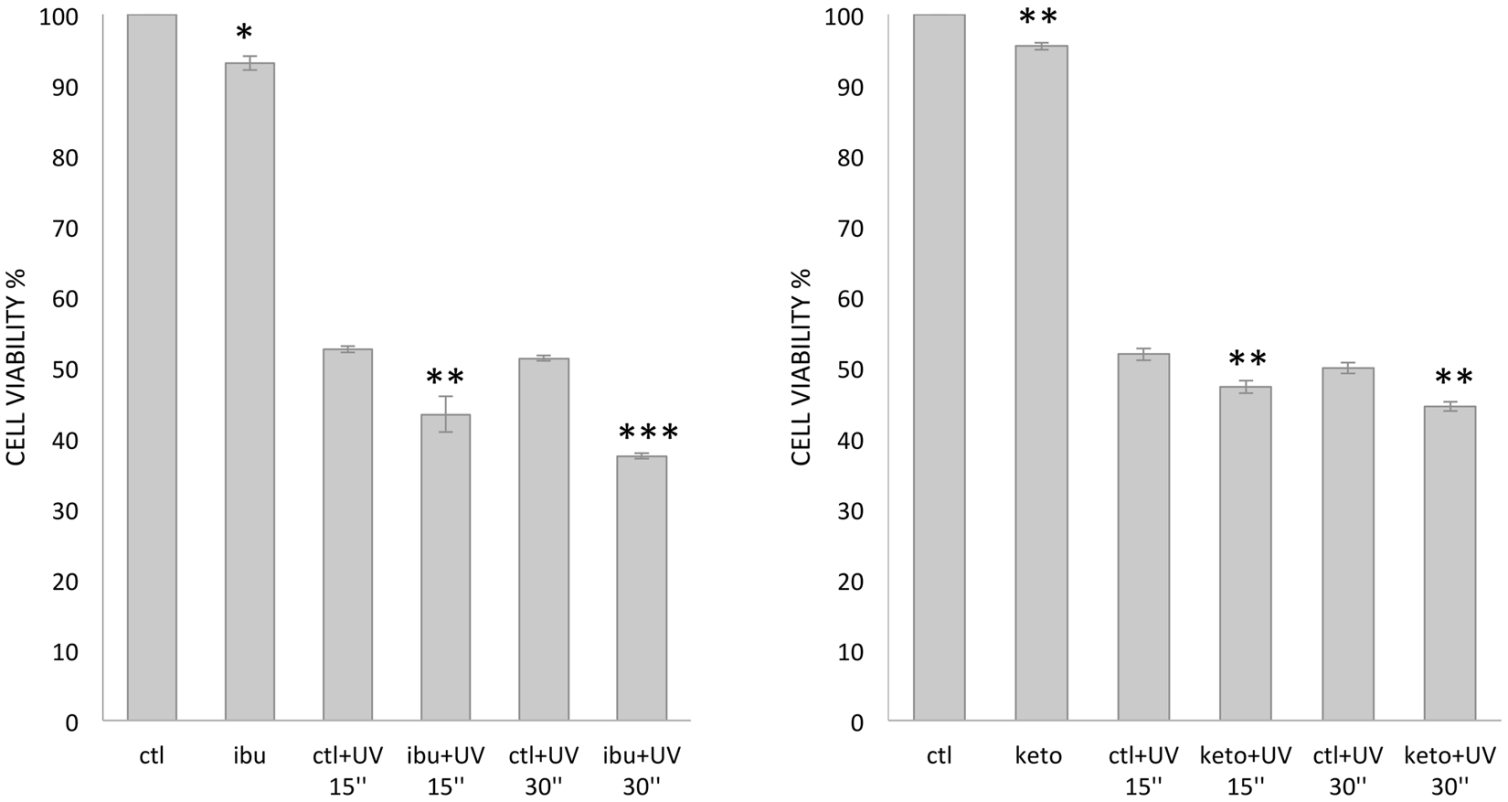
Ibuprofen (left) and ketoprofen (right) reduced cell viability upon UV radiation. Cell viability was assessed by MTT assays on MCF-7 cells untreated (ctl), or treated with 500 µM of ibuprofen/ketoprofen (ibu/keto), and/or irradiated with UV 15 s or 30 s. Cells were pretreated with 500 µM of ibuprofen or ketoprofen for 24 h before UV irradiation and reincubated for 17 h. Significance of untreated vs ibuprofen or ketoprofen treated cells are represented with * for P < 0.05, ** for P < 0.01, *** for P < 0.001.

As indicated in Fig. 2, while no significant difference of cell death was observed after 15 and 30 seconds of UV irradiation alone, pretreatment of MCF-7 cells with either ibuprofen or ketoprofen significantly affected the number of metabolically active cells in the control condition as well as after UV treatment.

**Figure 3:**
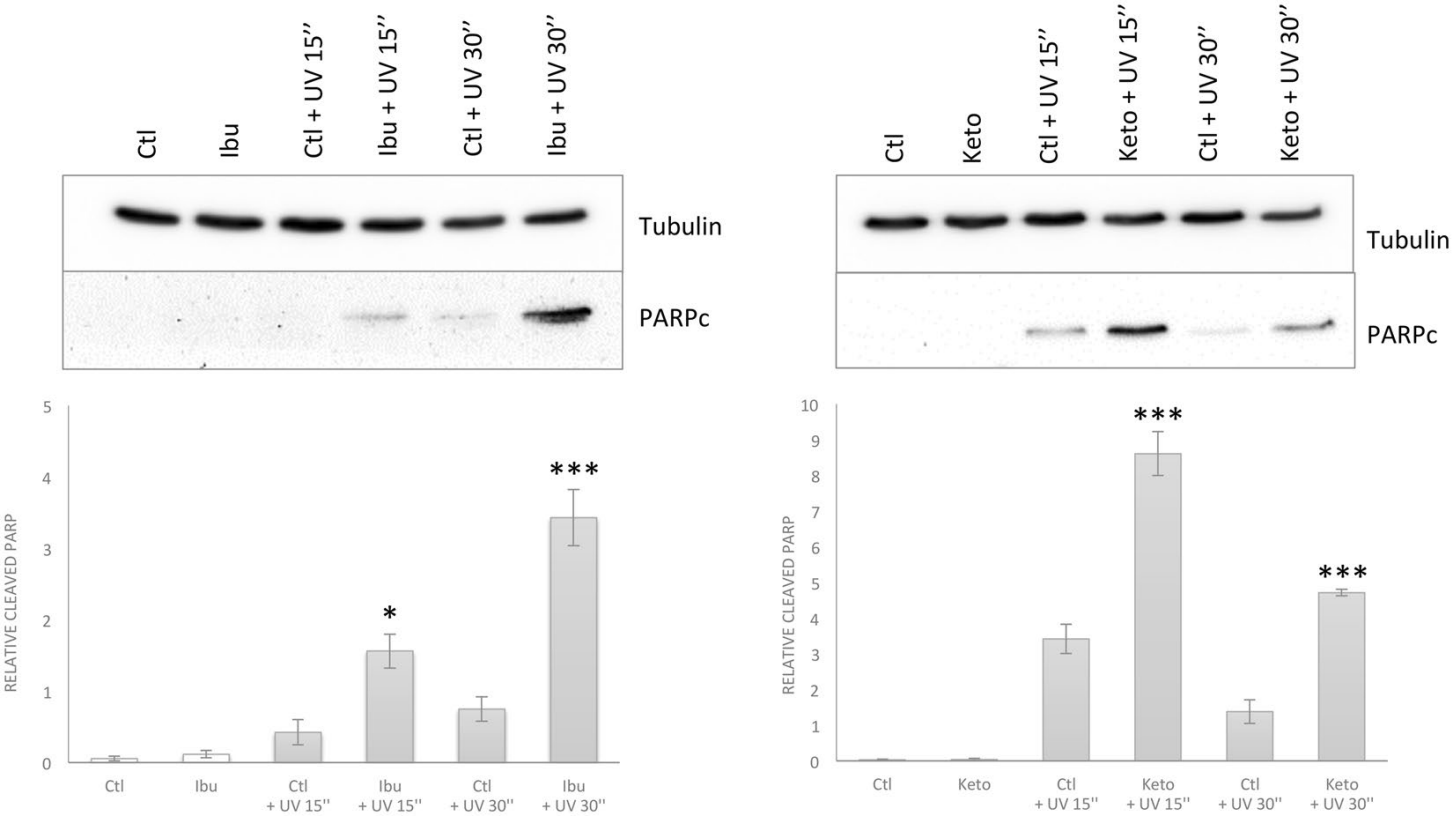
Ibuprofen (left) and ketoprofen (right) enhanced UV induced cell death. Cell death was evaluated by the detection of the cleaved PARP by western blotting on MCF-7 cells untreated (ctl) or treated with 500 µM of ibuprofen/ketoprofen (ibu/keto) as described in Fig. 2. 50 µg of total cell lysate was used. Detection of tubulin corresponds to the loading control. Significance of untretaed vs ibuprofen or ketoprofen treated cells are represented with * for P < 0.05, ** for P < 0.01, *** for P < 0.001. The full-length blots are presented in Supplementary Information (Figures S10 and S11).

To understand this effect, an analysis of cell death was performed by detecting the presence of cleaved PARP (Fig. 3), a marker of apoptosis. Our data showed a significant increase of cleaved PARP in MCF-7 cells pretreated with ibuprofen or ketoprofen compared to control cells, after UV irradiation. These data clearly indicate that ibuprofen and ketoprofen can by themselves influence cell metabolism without inducing cell death. However, a pretreatment of cells with either ibuprofen or ketoprofen before an exposure to UV increase cell death.

**Figure 4:**
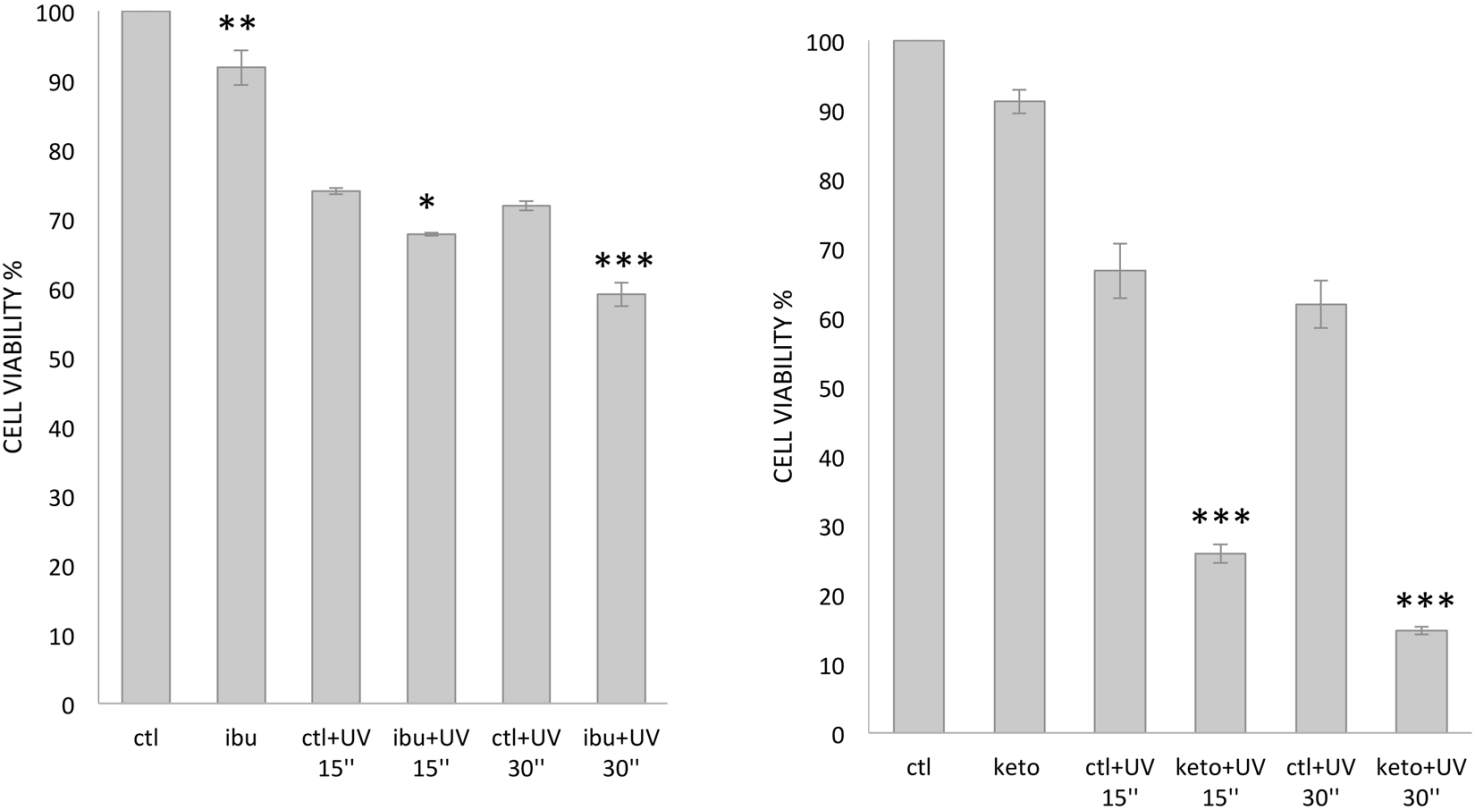
Ibuprofen and ketoprofen enhanced decrease of cell viability induced by UV light in MDA-MB231 cell lines. Cell viability was assessed by MTT assays as described in Fig. 2, and using a reincubation time of 6 h. Statistical significance for untreated vs ibuprofen or ketoprofen treated cells is represented with * for P < 0.05, ** for P < 0.01, *** for P < 0.001.

Our data were validated with the use of a second cell line. As shown in Fig. 4, the effects of ibuprofen and ketoprofen pretreatment on MDA-MB231 cells (i.e. before UV irradiation) were similar. Nevertheless, the decrease in cell viability upon UV irradiation was significantly more pronounced after ketoprofen pretreatment compared to ibuprofen pretreatment.

**Figure 5:**
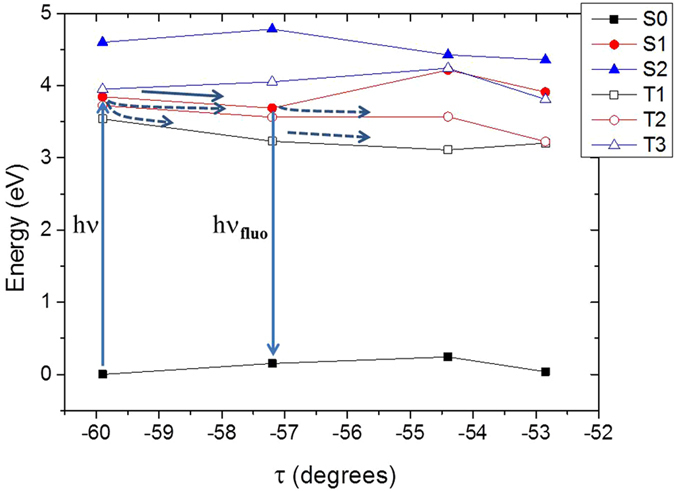
CASPT2//CASSCF MEP of ketoprofen in the S_1_ state, along the out-of-plane coordinate τ. TD-DFT and CASSCF results are provided as Supplementary Information and give the same global picture. After absorption to S_1_, different intersystem crossing pathways are open to populate the triplet manifold (dashed arrows), besides relaxation in S_1_ followed by fluorescence (solid arrows).

To rationalize these findings we analyze the properties of the two NSAIDs at the electronic and macromolecular level by molecular modeling and simulation. Both ibuprofen and ketoprofen absorb in the UVA region with maxima at 230 and 270 nm, respectively, as confirmed by theory and experiment (see Supplementary Information). This involves direct population of the optically bright π−π* S_1_ state for ibuprofen while for ketoprofen two possible pathways are open: population either of the brightest π−π* S_2_ state followed by fast internal conversion to the n-π* S_1_ state, or direct population of the latter. The electronic density reorganization upon excitation is depicted in Supplementary Information. In Fig. 5 we report the ketoprofen minimum energy path (MEP) along the dihedral angle τ (see Fig. 1a) in the photoexcited singlet S_1_ state. As it was the case for benzophenone^72^, we can underline the occurrence of two photophysical processes: delayed fluorescence from the S_1_ minimum and intersystem crossing.

**Figure 6:**
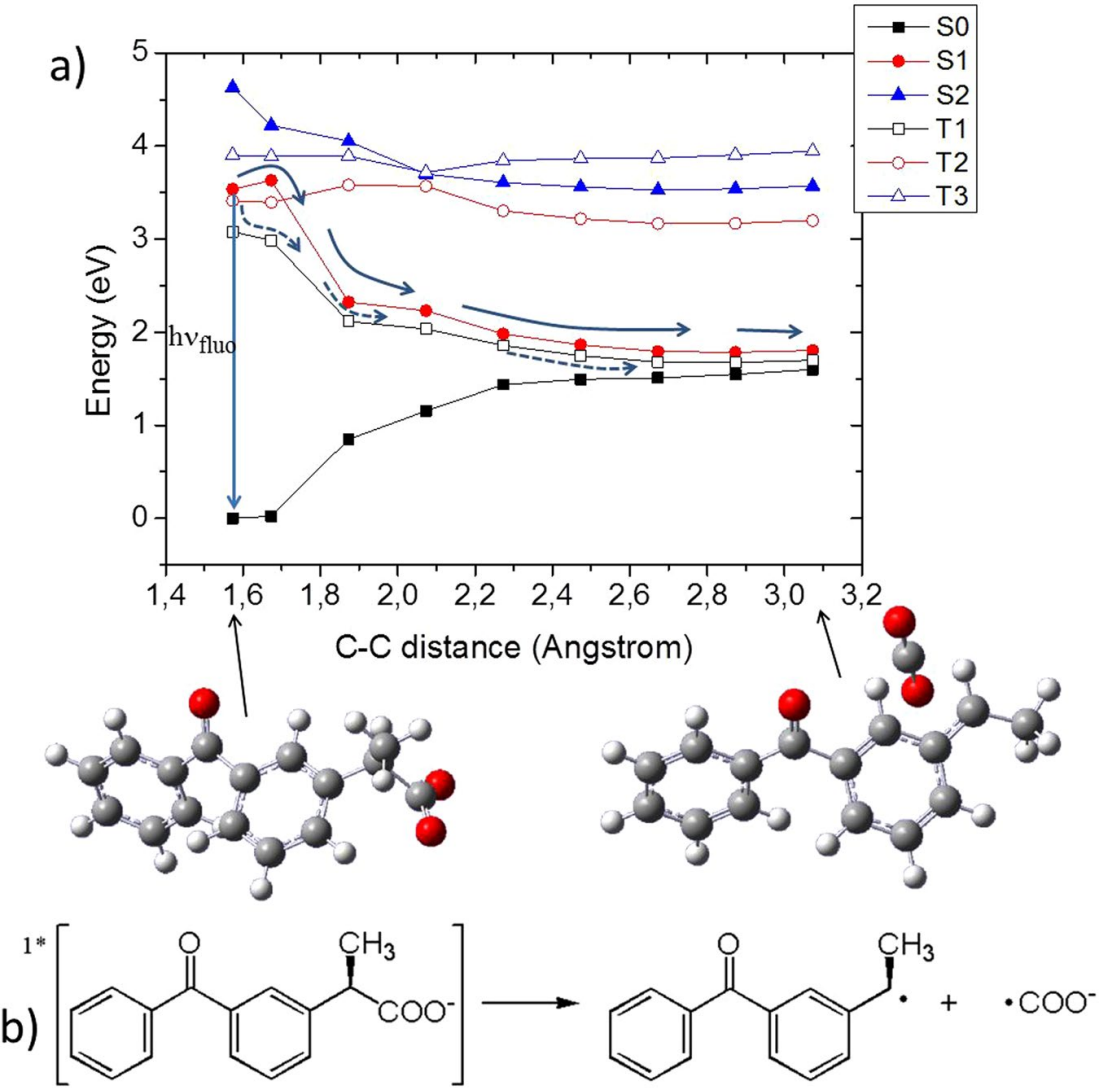
(**a**) CASPT2//CASSCF MEP of ketoprofen in the S_1_ and T_1_ state, shown as a function of the photo-dissociation coordinate. TD-DFT and CASSCF results are provided in Supplementary Information and give the same global picture. (**b**) Reactants and products are displayed, including the chemical formula.

Indeed, similar to the case of benzophenone, we recognize the presence of an extended region of quasi-degeneracy between S_1_ and the triplet T_2_ state, with the lowest-lying T_1_ state also being energetically available. This fact coupled to the relatively high spin-orbit coupling (around 20 cm^−1^) justifies the quasi-unitary population of the triplet manifold via a direct S_1_ →  T_1_ or indirect S_1_ → T_2_ → T_1_ mechanism as observed for benzophenone and confirmed via static^72^ and dynamic studies^73, 74^. However, a dissociative pathway for ketoprofen is also open as shown in Fig. 6.

Indeed, the dissociation of the COO^.-^ fragment can occur from the singlet manifold (S_1_), after overcoming a relative small barrier of about 0.1 eV, coherently with the presence of delayed fluorescence. Moreover, the lowest triplet state, T_1_, that can be populated via intersystem crossing, can also lead to the same reactive radical products via photodissociation (Fig. 6b).

**Figure 7:**
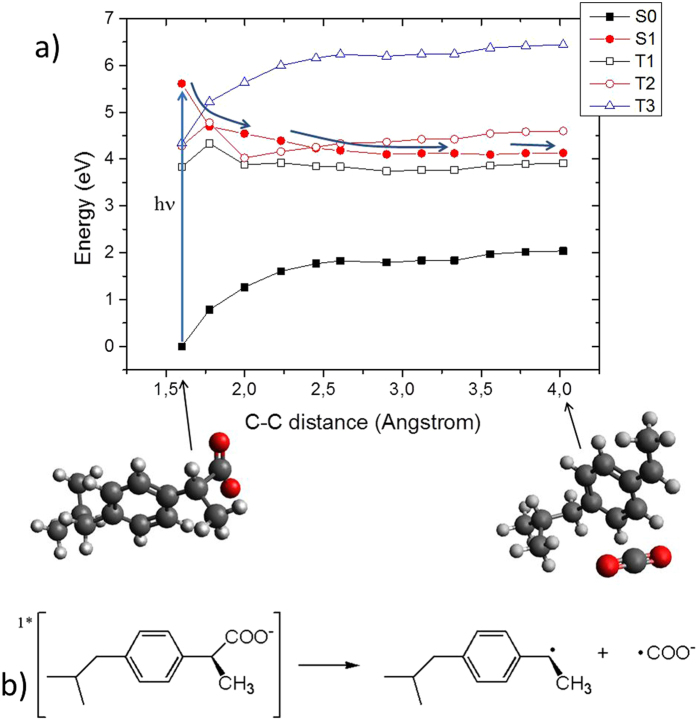
(**a**) CASPT2//CASSCF MEP of ibuprofen in the S_1_ state, shown as a function of the photo-dissociation coordinate. TD-DFT and CAS-SCF results are provided in Supplementary Information and give the same global picture. b) Reactants and products are displayed, including the chemical formula.

Ibuprofen on the other hand proceeds only via the dissociative pathways. In Fig. 7a we report the MEP along the photodissociative C-C distance and we observe that the S_1_ state proceeds to the barrierless dissociation of the chemical bond. This in turn will, again, produce the two highly reactive radical species, i.e. COO^.-^ and the residual of the core ibuprofen moiety as sketched in Fig. 7b. The direct comparison between ketoprofen and ibuprofen MEPs reveals small barrier vs. barrierless dissociation, which can be ascribed to the larger participation of the ibuprofen carbonyl group in the S_1_ electronic density reorganization (see Figures S7 and S9).

On the other hand the triplet manifold population can be ruled out for two reasons: first the barrierless potential energy surface observed herein points toward a ultrafast photodissociation, secondly even though the triplet states are energetically accessible, in particular T_1_, the spin-orbit coupling is extremely low all along the S_1_ potential energy surface never even reaching 1 cm^−1^ (see Figure S4).

The photo-toxicity of ibuprofen and ketoprofen is not only a photochemical intrinsic feature, but is also modulated by the strength and mode of interaction with biomolecules. Benzophenone was notably found to interact with DNA^71^ during extended simulation times, while calculations of the binding free-energy^75^ confirmed the existence of stable interaction modes. This fact assures the possibility, both from a spatial and temporal point of view, to trigger direct photo-damage of the nearby macromolecule. In order to assess the interaction with biological relevant structures, and in particular with DNA that is a common target for photosensitization, we performed classical molecular dynamics to evaluate the formation of persistent drug/DNA aggregates.

**Figure 8:**
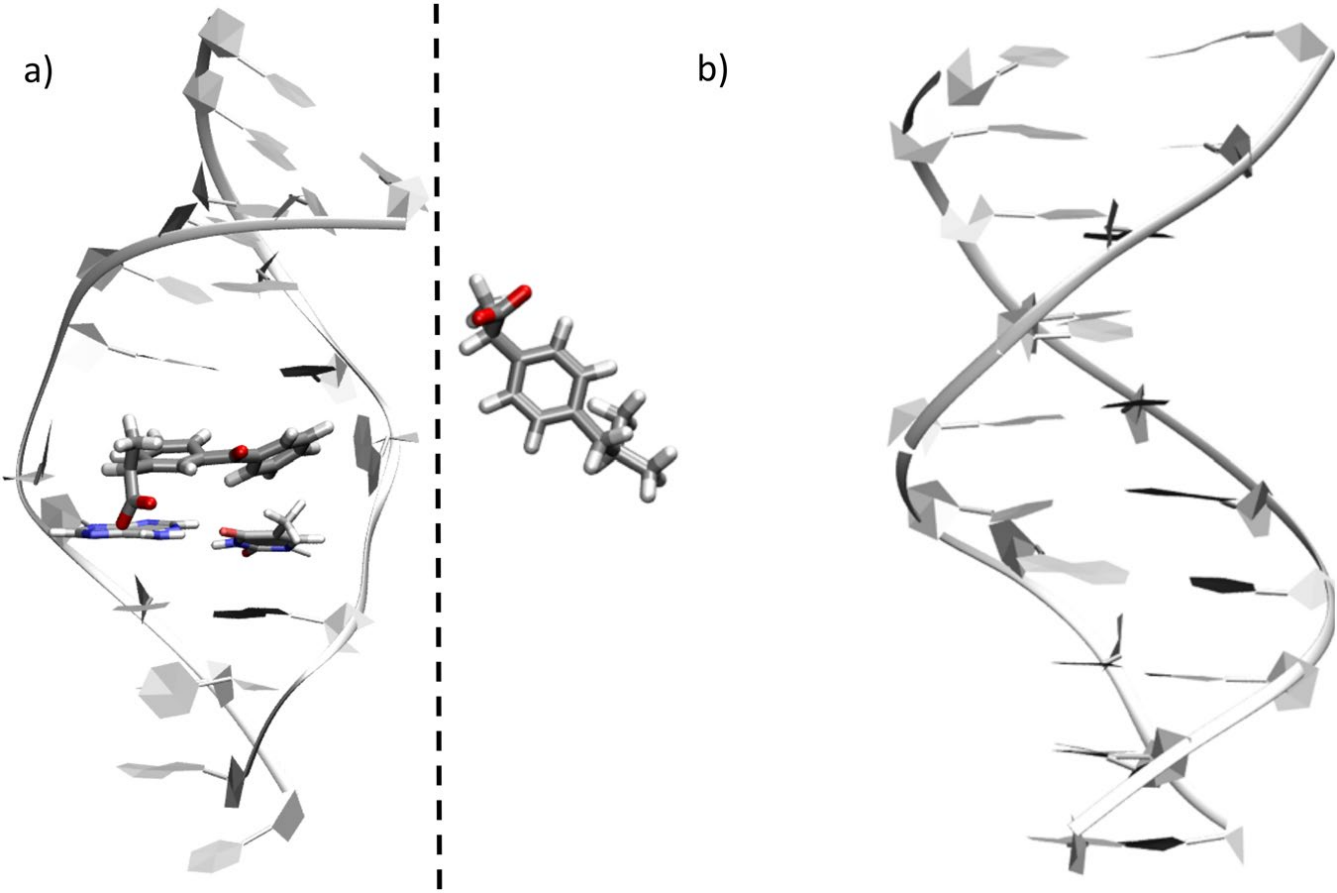
Representative structures extracted from the MD simulation of ketoprofen (**a**) and ibuprofen (**b**) interacting with a B-DNA duplex.

For ketoprofen, our simulations characterize a transient double insertion mode represented in Fig. 8, which persists a few nanoseconds. This lies in sharp contrast with the parent benzophenone whose DNA-bound stability exceeded 100 ns. Interactions with the minor groove are strongly destabilized, probably due to the presence of the negatively charged carboxylate group and consequently of repulsive interactions with the negatively charged phosphates of the DNA backbone. Hence, the intercalation or insertion of the conjugated rings in the hydrophobic DNA core appears as the most favored channel leading to aggregate formation and stabilization, as already observed for similar sensitizers^71, 76–78^. The DNA distortion upon interaction with the aryl ketone derivative remains local, as evidenced by the analysis of the global helical parameters. The more bulky character and the lower π-conjugation extent of ibuprofen, still bearing a negative charge, compromise the balance of non-covalent interactions. As a consequence, and differently from previous docking based studies^26^, much less persistent aggregates are observed.

## Discussion

The combined use of cellular biology assay and molecular simulations provides a global view of the photosensitization activity of two extremely common drugs. It is evident that the combined exposure to UV light and ibuprofen or ketoprofen induces a marked decreased of metabolically active cells as well as an increase in cell death level. The results were confirmed on two different cell lines. It has to be noticed that these results were obtained on two different cell lines, which are known to be more resistant than normal cells. Thus, effects of ibuprofen and ketoprofen could be even more deleterious in normal cell lines. However, ketoprofen and ibuprofen display markedly different behavior in the observed effects over the two lines, hence suggesting different modes of action at the molecular level. On the other hand the absence of differential effects after 15 and 30 seconds irradiation is indicative of the absence of dose response. However, usually the study of the dose response is performed varying the intensities of the source rather then the exposure time, and in addition our UV dose (0,5mJ/s/cm^2^) was deliberatively kept low to demonstrate the impact of NSAIDs even at low irradiation. The absence of a time response is also coherent with the result obtained by Panno *et al*.^79^.

Indeed two distinct pathways have been evidenced for the two drugs: while ibuprofen irradiation triggers photodissociation of the carboxylic moiety from the singlet manifold, ketoprofen populates the triplet manifold via intersystem crossing, and can experience dissociation both from S_1_, overcoming a relative small barrier, and barierlessly from T_1_. In the triplet manifold ketoprofen may provoke all the diverse sensitization effects characteristic of its parent compound benzophenone^35^, and in particular triplet-triplet energy transfer to DNA bases or singlet oxygen production. Furthermore, the photodissociation will produce highly reactive radical species able to react with biological macromolecules. In addition, and coherently with our scenario, photodissociation of triplet state protonated ketoprofen^51, 52^, following proton transfer from the carboxylic to the carbonyl group has been reported^80^. However, differently from benzophenone, and due to the influence of the negative charge of its salt form, ketoprofen only exhibits metastable interactions with DNA. Hence we may conclude that its predominant mode of action will be either through the production of singlet oxygen or via the generation of the reactive radical species that do not necessitate a direct coupling with the DNA bases. For ibuprofen on the other hand only the photodissociative pathway from S_1_ is open. Hence, it only produces the two reactive radical species that may induce considerable damages to biological macromolecules.

Speaking about the photosensitization exerted through the radical moieties, and in the case of DNA one may hypothesize the production not only of nucleobase oxidation but also of strand breaks that are known to be extremely toxic to the cells. Hence, the photosensitization has to be considered as non-selective such that it can affect not only DNA but also lipid membranes and proteins. Nevertheless, the presence of metastable interactions, coupled with the generation of highly reactive intermediates, would increase the propensity to induce DNA sensitization.

From a purely mechanistic point of view, ibuprofen and ketoprofen photophysics and photochemistry deserve to be regarded as most likely to induce considerable damages to DNA and other biological structures. Indeed ibuprofen barrierless ultrafast dissociation could be more efficient than the triplet population of ketoprofen or its dissociation from the singlet manifold due to the small energy barrier. However, one has to take into account that the ibuprofen absorption maximum is found at 230 nm and only the absorption tail covers the UVA window, *i*.*e*. the portion of the UV spectrum not filtered by the upper atmospheric ozone layers. Consequently, only a limited amount of ibuprofen molecules will indeed be excited by UVA light and hence the overall photosensitization rate will be less pronounced.

To summarize, our study has clearly shown, and firmly rationalized, the photosensitization efficiency of the two NSAIDs, correlating these to a significant decrease of metabolically active cell number and the consequent increase in cell death. However, UV light is efficiently screened by the skin and its penetration length is strongly reduced. Hence, oral intake of ibuprofen or ketoprofen drugs can be seen as safe from a photosensitization point of view. However, both drugs are also used in topical preparations, as creams, gels or patches, to be applied directly to the skin, *i*.*e*. in parts of the body directly exposed to UV light. Hence, care should be taken in the case of exposure to sunlight or phototherapy, in conjunction with the simultaneous application of topical ibuprofen or ketoprofen based formulations.

## Electronic supplementary material

Supplementary Information
