# Supplementary material for: Ibuprofen and ketoprofen potentiate UVA-induced cell death by a photosensitization process
Source: Sci Rep. 2017 Aug 21;7:8885. doi: 10.1038/s41598-017-09406-8 (PMC5566383; doi:10.1038/s41598-017-09406-8)
Supplement: Supplementary file 1 — Supplementary Information [file 41598_2017_9406_MOESM1_ESM.pdf]

Ibuprofen and ketoprofen potentiate UVA-induced cell death by a photosensitization process

Emmanuelle Bignon,[1,2,†] Marco Marazzi,[3,4,†] Vanessa Besancenot,[5] Hugo Gattuso,[3,4] Guillaume Drouot,[5,6] Christophe Morell,[1] Leif Eriksson,[6] Stephanie Grandemange,[5,*] Elise Dumont,[2,*] Antonio Monari[3,4,*]

[1]Institut des Sciences Analytiques, UMR 5280, Université de Lyon1 (UCBL) CNRS, ENS Lyon, Lyon, France

[2]Université de Lyon, ENS de Lyon, CNRS, Université Lyon 1, Laboratoire de Chimie, F69342, Lyon, France.

[3]Theory-Modeling-Simulation, Université de Lorraine − Nancy, SRSMC Boulevard des Aiguillettes, Vandoeuvre-lès-Nancy, Nancy, France

[4]Theory-Modeling-Simulation, CNRS, SRSMC Boulevard des Aiguillettes, Vandoeuvre-lès-Nancy, Nancy, France. [5]Université de Lorraine Nancy and CNRS, CRAN, Vandeouvre-lès-Nancy, France.

[6]Department of Chemistry & Molecular Biology, University of Göthenburg, Medicinaregatan 9 c, 40530 Göteborg, Sweden

Content

1. Computational details
2. TD-DFT benchmark
3. Experimental and theoretical absorption spectra in water
4. Molecular orbitals and minimum energy paths at different levels of theory
5. Full Blots (see Figure 3 in Main Text)

1. Computational details

Concerning molecular dynamics, for each system, 19 potassium cations and an orthorhombic TIP3P water box were added, resulting in ~25000 atoms in total. Then, the geometry was minimized using 5000 steps with the steepest descent algorithm followed by 5000 using the conjugate gradient. The temperature was raised from 0 to 300 K in a 30 ps heating run. The 300 K temperature value was kept constant during the remaining of the simulation using the Langevin thermostat with a collision frequency γln of 1 ps^{-1}. The system was then relaxed in NTP conditions during a 1 ns equilibration step. Finally, a production stage was performed to sample the conformational space of each system during 300 ns for inserted starting structures and only 100 ns for minor groove binding initial positions since the latter were not stable due to the repulsion between negatively charged DNA phosphates and the drugs' carboxylate.

Concerning quantum chemistry calculations, for both ibuprofen and ketoprofen, Multi State (MS)-CASPT2//State Average (SA)-CASSCF calculations were performed, including the three lowest-energy singlet (S_0, S_1, S_2) and triplet (T_1, T_2, T_3) states. When applying the CASPT2 energy correction on top of the CASSCF calculation, an imaginary shift of 0.2 and no IPEA shift were included.

2. TD-DFT benchmark

A benchmark study to select the appropriate DFT functional was performed on ibuprofen. Especially, the static absorption spectrum – *i.e.* the absorption spectrum calculated for the ground state (S_0) optimized molecule – is shown in Table S1. Solvent (water) effects are taken into account by PCM (see main text).

Table S1. Ibuprofen absorption spectra calculated at TD-DFT level for different functionals. The 6-311+G** basis set is used for all cases. Energy values are given in eV and (nm). In the last row, the CASPT2/ANO-L-VDZP results are shown for comparison.

Functional/method	S_0–T_1	S_0–T_2	S_0–T_3	S_0–S_1
**CAM-B3LYP**	3.44 (361)	4.39 (282)	4.57 (272)	5.60 (222)
**B3LYP**	3.64 (340)	4.22 (294)	4.43 (280)	4.96 (250)
**BLYP**	3.65 (339)	3.68 (337)	3.80 (326)	4.03 (308)
**PBE0**	3.48 (356)	4.25 (292)	4.45 (279)	5.11 (243)
**PBE**	3.59 (346)	3.61 (343)	3.71 (334)	3.96 (313)
**BP86**	3.61 (343)	3.64 (341)	3.75 (330)	4.01 (309)
**CASPT2**	3.83 (324)	4.28 (290)	4.34 (286)	5.61 (221)

As it can be seen, the relative energy of the bright singlet state that is populated after irradiation (S_0–S_1) is can be highly dependent on the selected functional. As compared to CASPT2/ANO-L-VDZP calculations, we decided to select the CAM-B3LYP functional, being only 0.01 eV blue shifted. For all functionals, three triplet states lie below S_1, as suggested also by the CASPT2 reference calculation.

3. Experimental and theoretical absorption spectra in water

Absorption spectra were calculated for ibuprofen and ketoprofen, also including the dynamical effect: after ground state (S_0) optimization, the phase space around the S_0 minimum structure was sampled through a Wigner distribution.[S1,S2] As input, the vibrational frequencies and the corresponding normal mode vectors were provided by a frequency calculation at the CAM-B3LYP/6-311+G** level of theory. Further, an excited states calculation of the lowest-energy five singlet states (S_0 to S_4) was performed for twenty random structures obtained from the Wigner distribution. The absorption spectrum has been calculated as the Gaussian convolution (FWHM = 0.4) of the linear transitions for all the structures. Solvent (water) effects are taken into account by PCM (see main text).

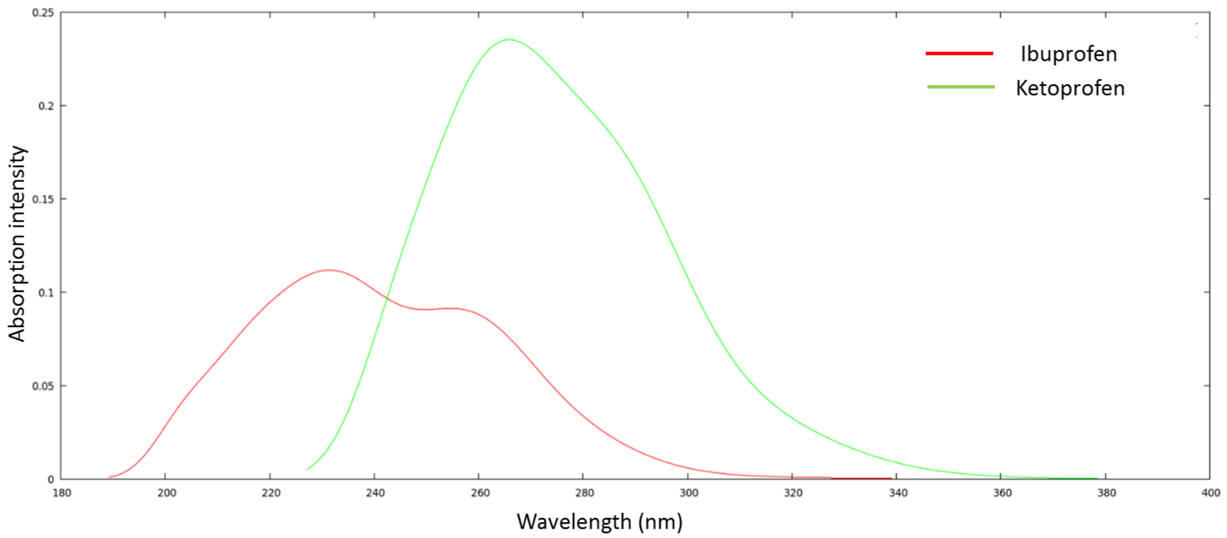

Figure S1. Ibuprofen (red) and ketoprofen (green) calculated absorption spectra in water.

When compared to the experimental spectra recorded in our laboratory (Figure S2) and to the available experimental data (Figure S3, data taken from ref. S3 for ibuprofen and ref. S4 for ketoprofen), we can observe how the simulated spectra are in good agreement with the experience, also reproducing the overall spectrum shape, even though slightly overestimating the absorption intensity around 260 nm for ibuprofen.

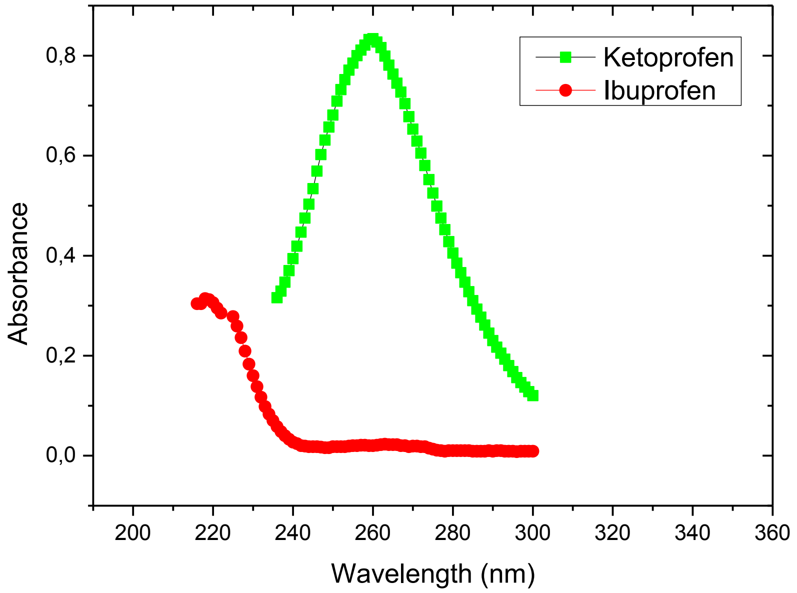

Figure S2. Ibuprofen (red) and ketoprofen (green) experimental absorption spectra in water. Data recorded in our laboratory at the following concentrations, for solubility issues: 2 mM (ibuprofen), 500 µM (ketoprofen).

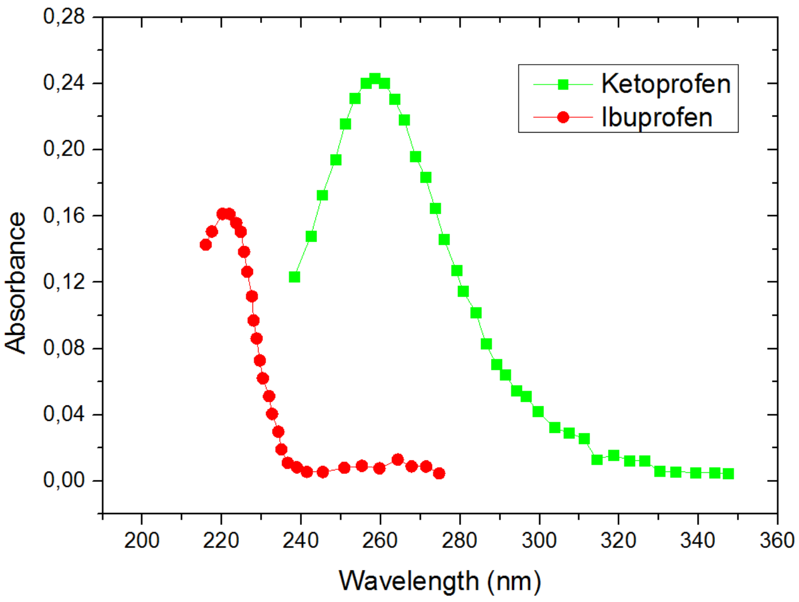

Figure S3. Ibuprofen (red) and ketoprofen (green) experimental absorption spectra in water. Data taken from refs. S3 and S4, followed by absorbance normalization.

(S1) J. P. Dahl, M. Springborg *J. Chem. Phys.* **1988**, *88*, 4535-4547.

(S2) R. Schinke *Photodissociation Dynamics: Spectroscopy and Fragmentation of Small Polyatomic Molecules* (Cambridge University Press), 1995.

(S3) J. L. Packer, J. J. Werner, D. E. Latch, K. McNeill, W. A. Arnold *Aquat. Sci.* **2003**, *65*, 342-351.

(S4) S. Monti, S. Sortino, G. De Guidi, G. Marconi *New J. Chem.* **1998**, 599-604.

4. Molecular orbitals and minimum energy paths at different levels of theory

In Figure S3 the minimum energy paths of ibuprofen and ketoprofen at TD-DFT and CASSCF levels are shown.

In Figure S4 it is shown the spin-orbit coupling calculated for ibuprofen along the photo-dissociation coordinate, between S_1 and the three lowest-energy triplet states (T_1, T_2 and T_3). As it can be seen, the values are really low in all cases, never reaching 1 cm^{-1}. Therefore, singlet-to-triplet intersystem crossing mechanisms can be discarded. On the other hand, the spin-orbit coupling calculated for ketoprofen (in Franck–Condon and S_1 minimum) confirmed its benzophenon-like behavior, being constantly of *ca.* 20 cm^{-1}.

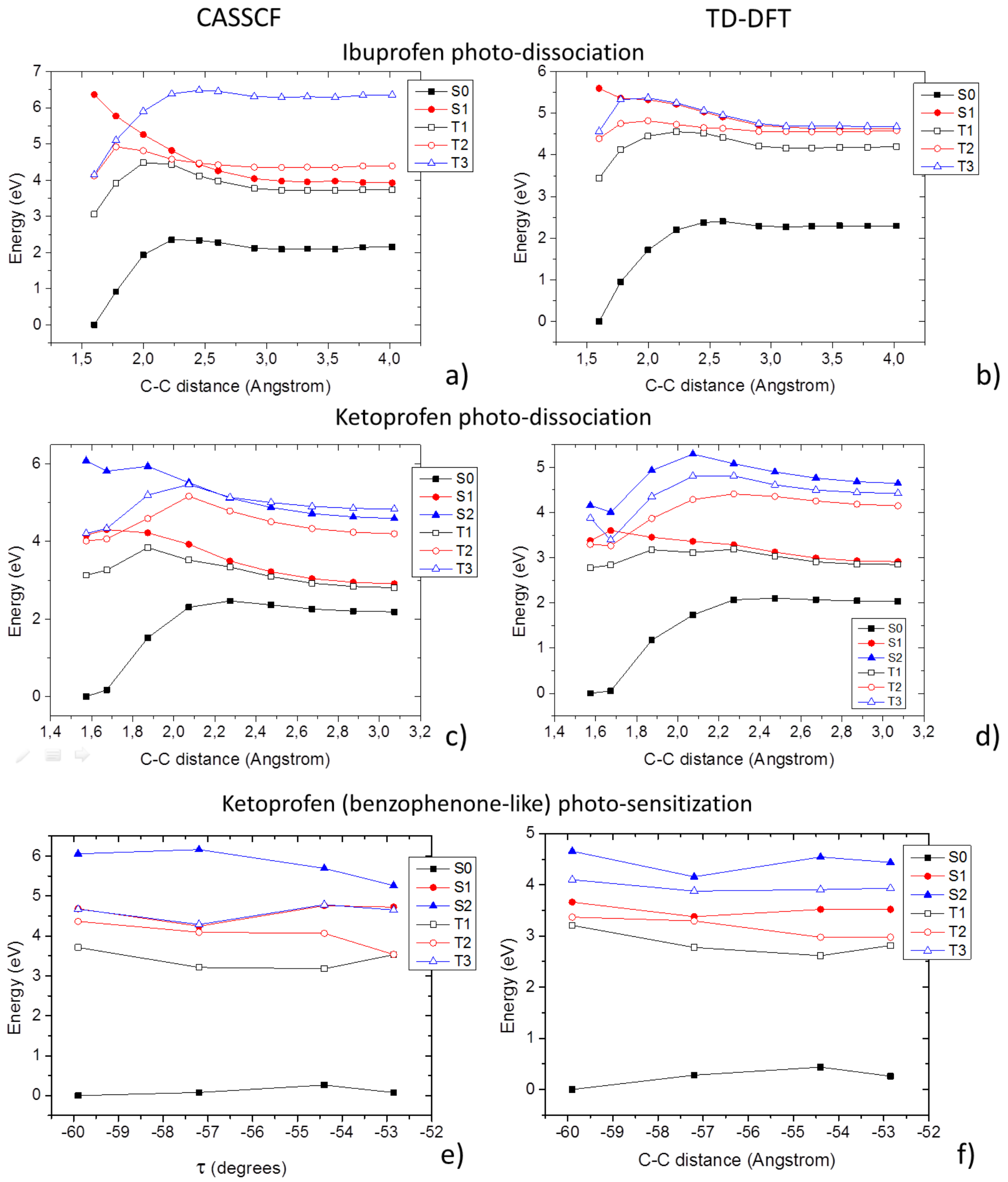

Figure S4. Ibuprofen (a,b) and ketoprofen (c,d,e,f) minimum energy paths at CASSCF/ANO-L-VDZP (a,c,e) and CAM-B3LYP/6-311+G** (b,d,f) levels of theory.

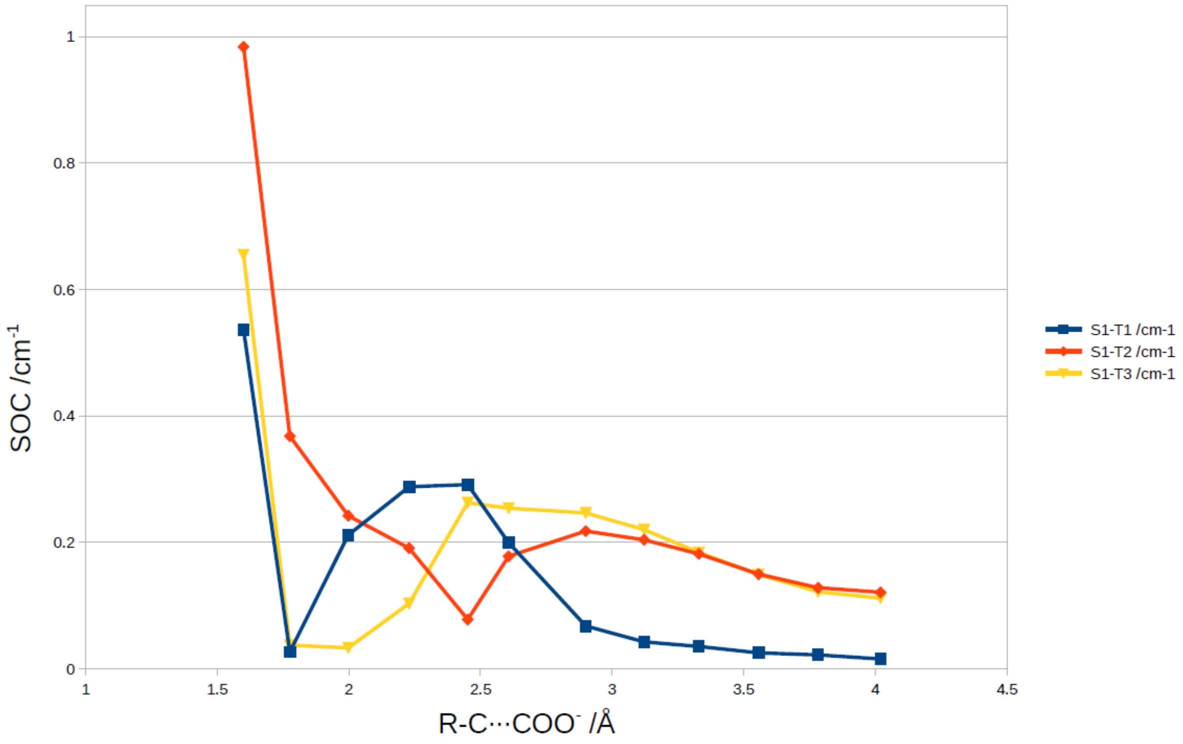

Figure S5. Ibuprofen spin-orbit coupling (SOC) along the mimimum energy path, shown as a function of the photodissociation coordinate.

The molecular orbitals of the studied systems are shown in Figures S6, S7 (CASSCF level), S8 and S9 (TD-DFT level).

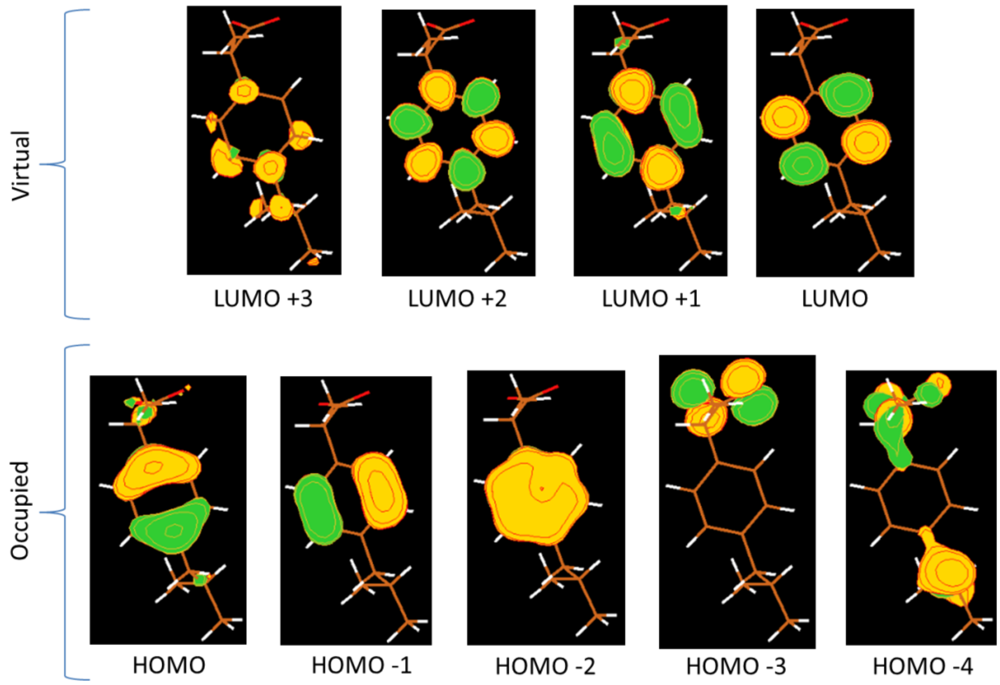

Figure S6. Ibuprofen CASSCF/ANO-L-VDZP molecular orbitals.

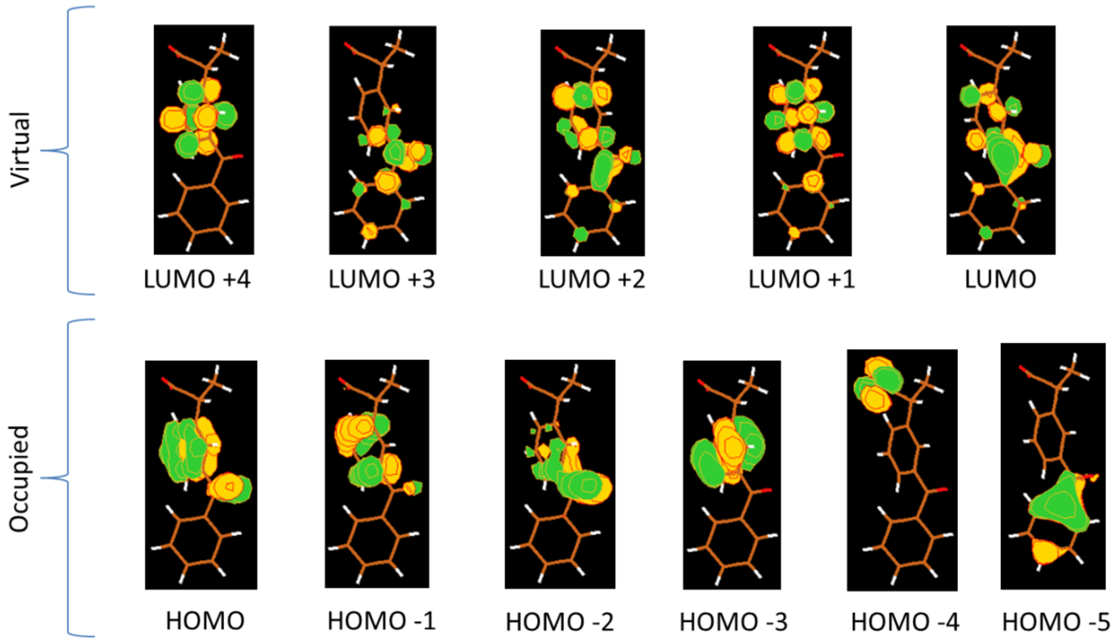

Figure S7. Ketoprofen CASSCF/ANO-L-VDZP molecular orbitals.

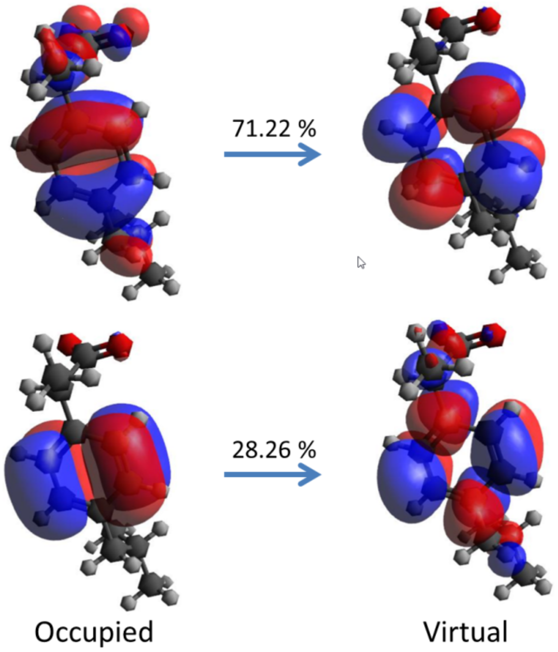

Figure S8. Ibuprofen CAM-B3LYP/6-311+G** natural transition orbitals (NTOs). The relative weight of the principal square singular values are shown on the arrow.

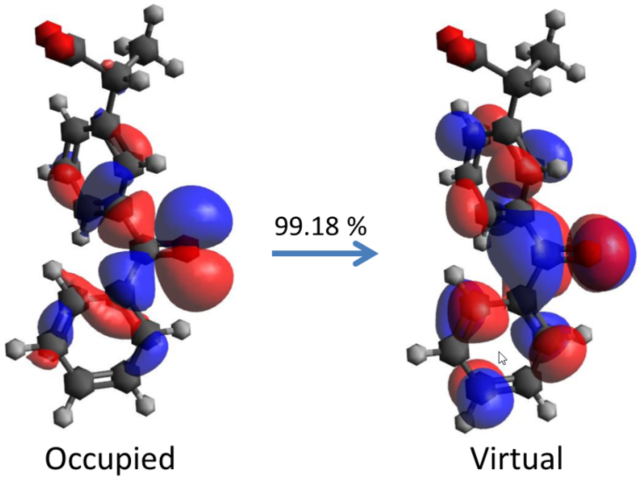

Figure S9. Ketoprofen CAM-B3LYP/6-311+G** natural transition orbitals (NTOs). The relative weight of the principal square singular value is shown on the arrow.

As it can be seen, CASSCF and TD-DFT orbitals largely match in shape. Therefore, we can confirm that both level of theory can be used to describe the photochemistry of both NSAIDs.

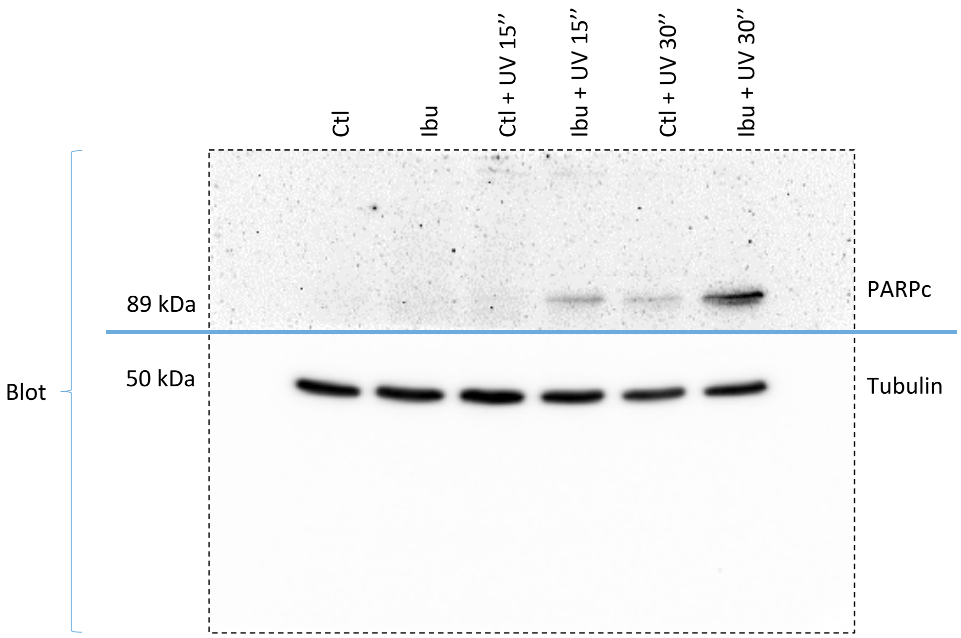

Figure S10. This figure represents the full-length blot from Figure 3 in the main text. The blot was cropped in two parts (as represented by the blue line). The two parts of the blot are from the same gel. The upper part was used to detect the cleaved PARP protein and the lower part was used to detect tubulin protein.

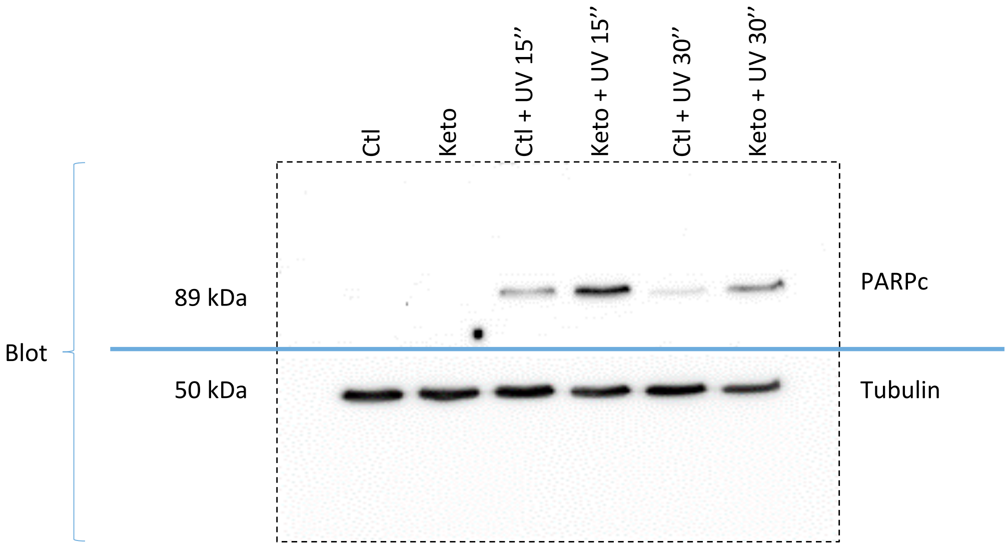

Figure S11. This figure represents the full-length blot from Figure 3 in the main text. The blot was cropped in two parts (as represented by the blue line). The two parts of the blot are from the same gel. The upper part was used to detect the cleaved PARP protein and the lower part was used to detect tubulin protein.
